# Author Correction: Epstein–Barr virus-encoded microRNA BART1 induces tumour metastasis by regulating PTEN-dependent pathways in nasopharyngeal carcinoma

**DOI:** 10.1038/s41467-020-17048-0

**Published:** 2020-07-06

**Authors:** Longmei Cai, Yanfen Ye, Qiang Jiang, Yuxiang Chen, Xiaoming Lyu, Jinbang Li, Shuang Wang, Tengfei Liu, Hongbing Cai, Kaitai Yao, Ji-Liang Li, Xin Li

**Affiliations:** 1 Cancer Research Institute, Southern Medical University, Guangzhou, 510515 China; 2 Radiation Department, The First Affiliated Hospital, Wannan Medical College, Wuhu, 241001 China; 3 Department of Pathology, Basic Medical College, Southern Medical University, Guangzhou, 510515 China; 4 School of Traditional Chinese Medicine, Southern Medical University, Guangzhou, 510515 China; 5 School of Biotechnology, Southern Medical University, Guangzhou, 510515 China; 6 Molecular Oncology Laboratories, Department of Oncology, Weatherall Institute of Molecular Medicine, University of Oxford, John Radcliffe Hospital, Oxford, OX3 9DS UK

Correction to: *Nature Communications* 10.1038/ncomms8353, published online 2 July 2015.

This Article contains an error in Fig. 7. In Fig. 7a, the image for in-b1-5p is inadvertently duplicated from the in-b1-3p image.



When preparing the correction the authors noticed that the images incorrectly represent an invasion experiment rather than a migration experiment. New images have been provided for both in-b1-5p and in-b1-3p.



Representative images for three independent migration assays have been provided as Supplementary Fig. 1.



The error has not been corrected in the PDF or HTML versions of the Article.

